# Supplementary material for: Differentiation and Identification of Endophytic Bacteria from Populus Based on Mass Fingerprints and Gene Sequences
Source: Int J Mol Sci. 2023 Aug 30;24(17):13449. doi: 10.3390/ijms241713449 (PMC10487577; doi:10.3390/ijms241713449)
Supplement: Supplementary file 1 [file ijms-24-13449-s001.zip › Supplementary Figure and Table.pdf]

Supplementary Material

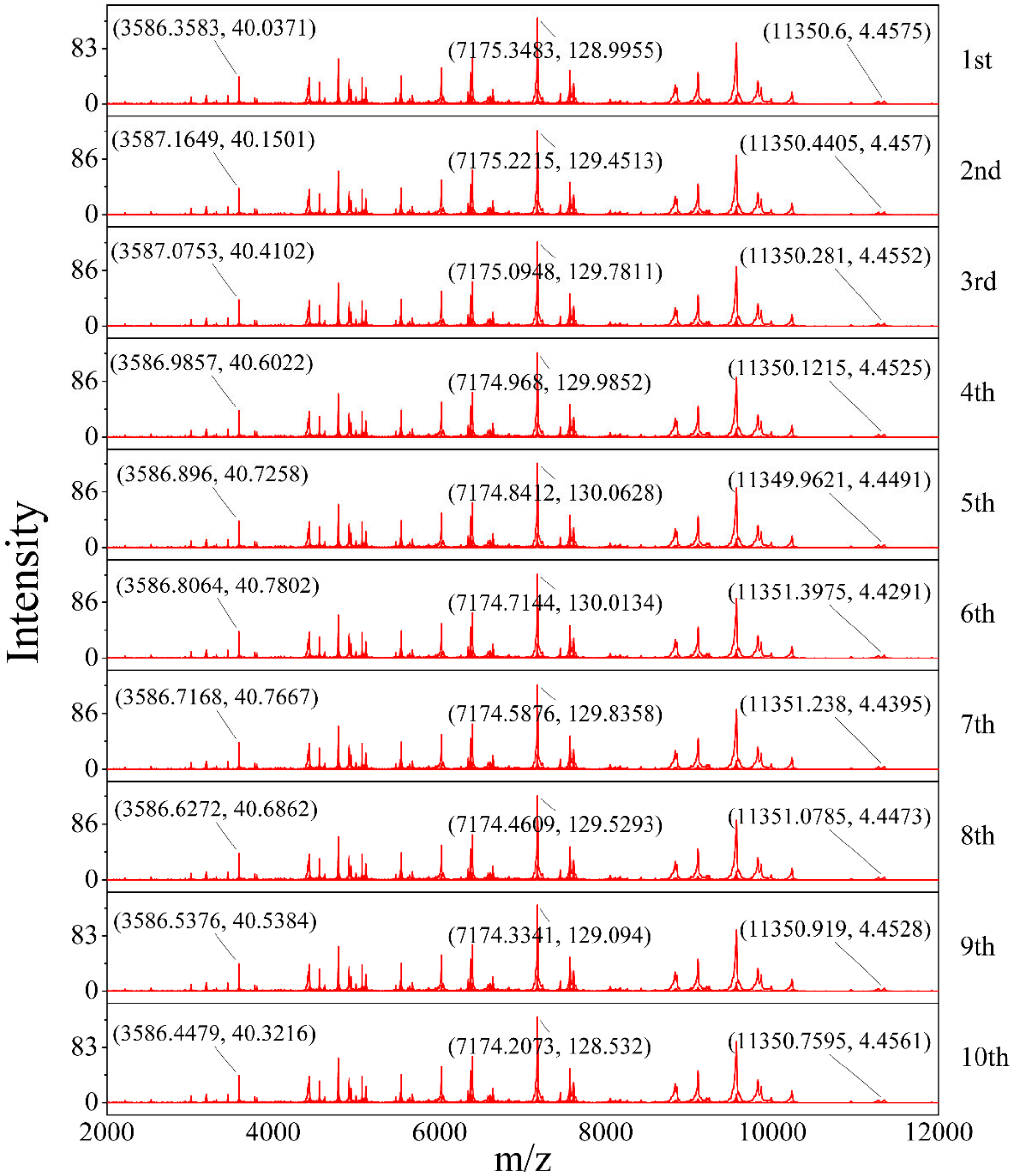

Figure S1. Verification experiments for optimized method reproducibility

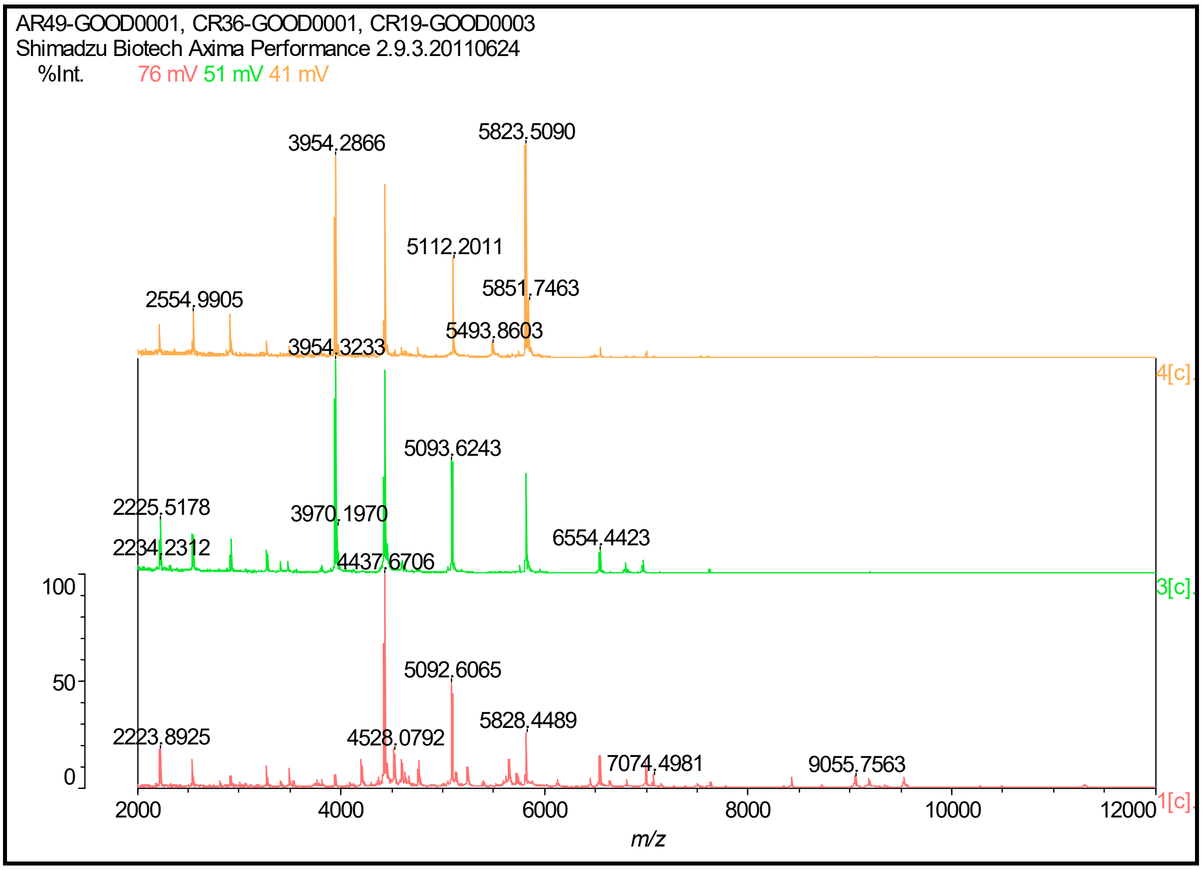

Figure S2. Comparison of proteins mass fingerprints of three Gram-positive endophytic bacteria, *Microbacterium testaceum* AR49, CR36 and CR19 from the top down

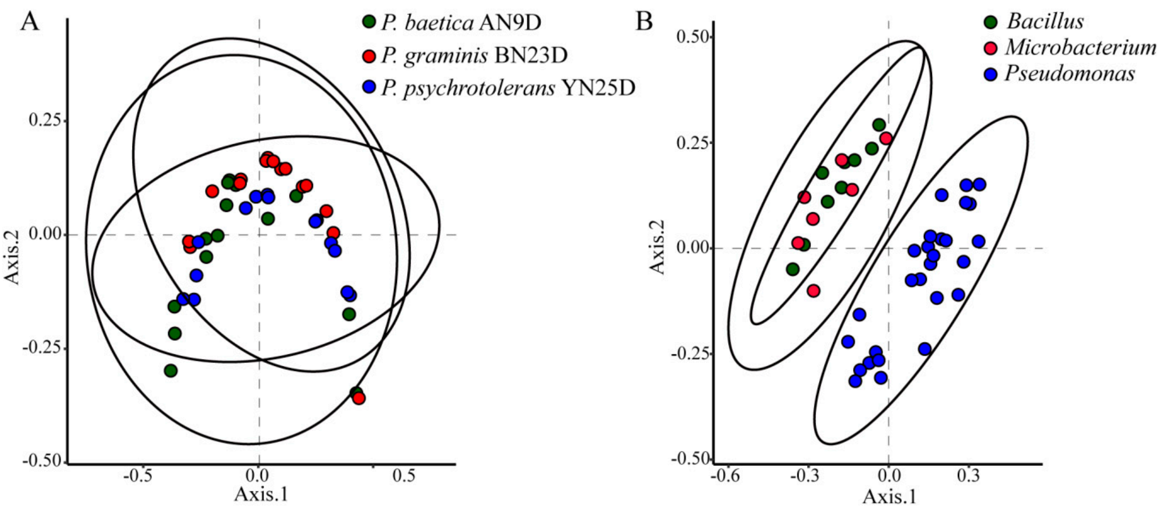

Figure S3. Principal co-ordinates analysis (PCoA) based on Bray-Curtis dissimilarities demonstrated the distance of MALDI-TOF MS profiles using raw data. A. each fifteen biological replicates of *P. baetica* AN9D, *P. graminis* BN23D and *P. psychrotolerans* YN25D. B. 43 species endophytic bacteria belong to *Bacillus*, *Microbacterium* and *Pseudomonas*.

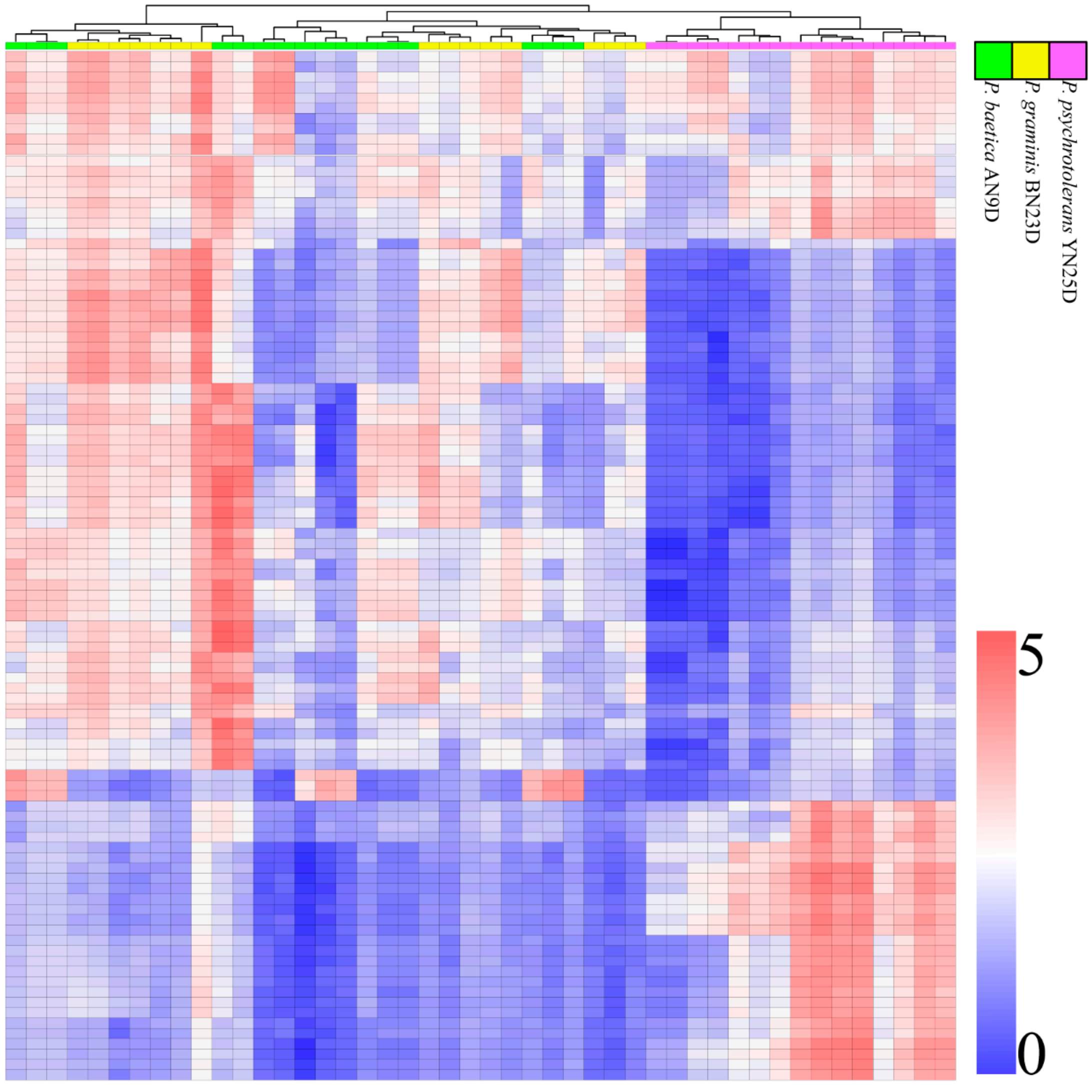

Figure S4. Clustering heatmap of the raw data, the data including 15 biological repeat of *Pseudomonas baetica* AN9D, 15 biological repeat of *P. graminis* BN23D and 16 biological repeat of *P. psychrotolerans* YN25D.

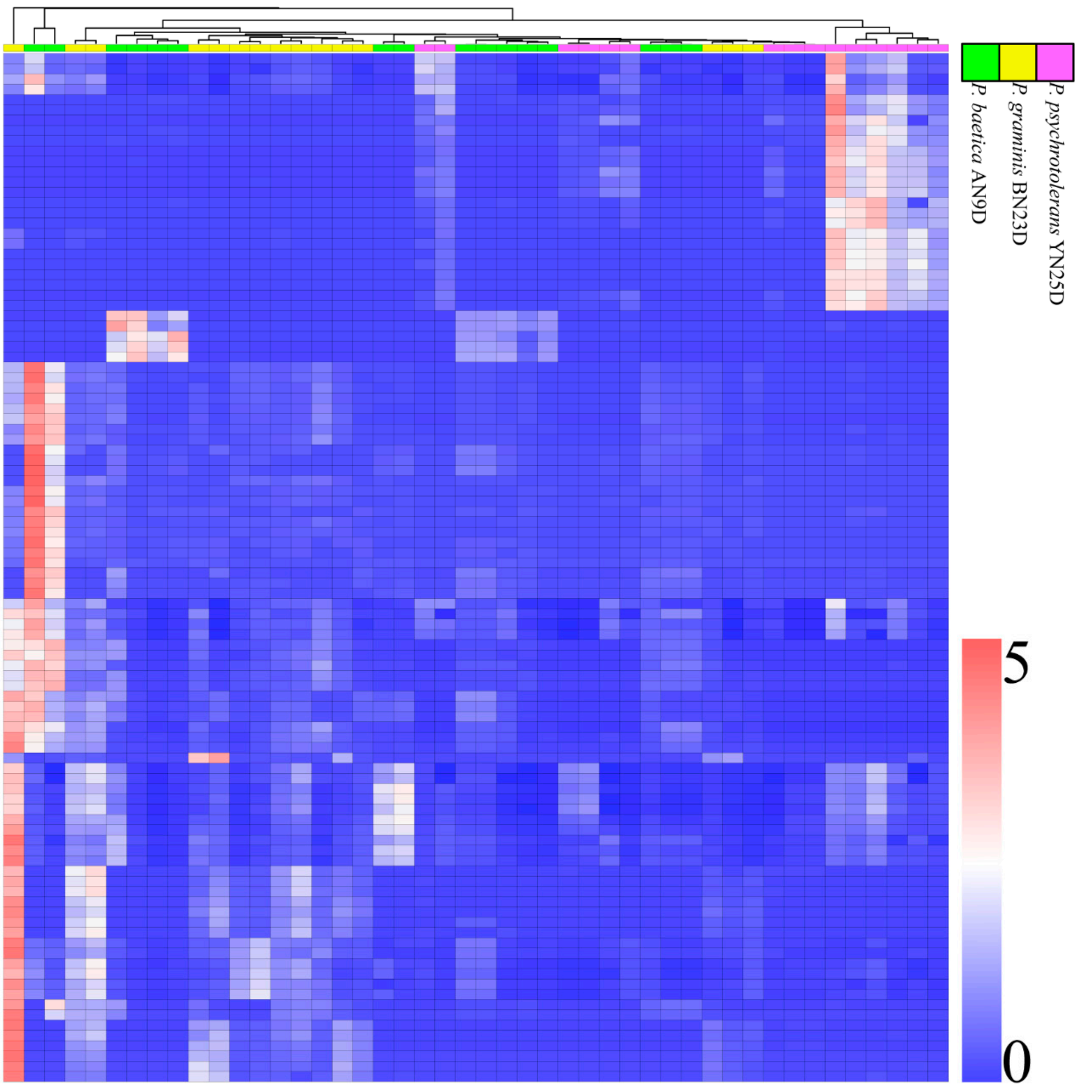

Figure S5. Clustering heatmap of the de-nosed data, the data including 15 biological repeat of *Pseudomonas baetica* AN9D, 15 biological repeat of *P. graminis* BN23D and 16 biological repeat of *P. psychrotolerans* YN25D.

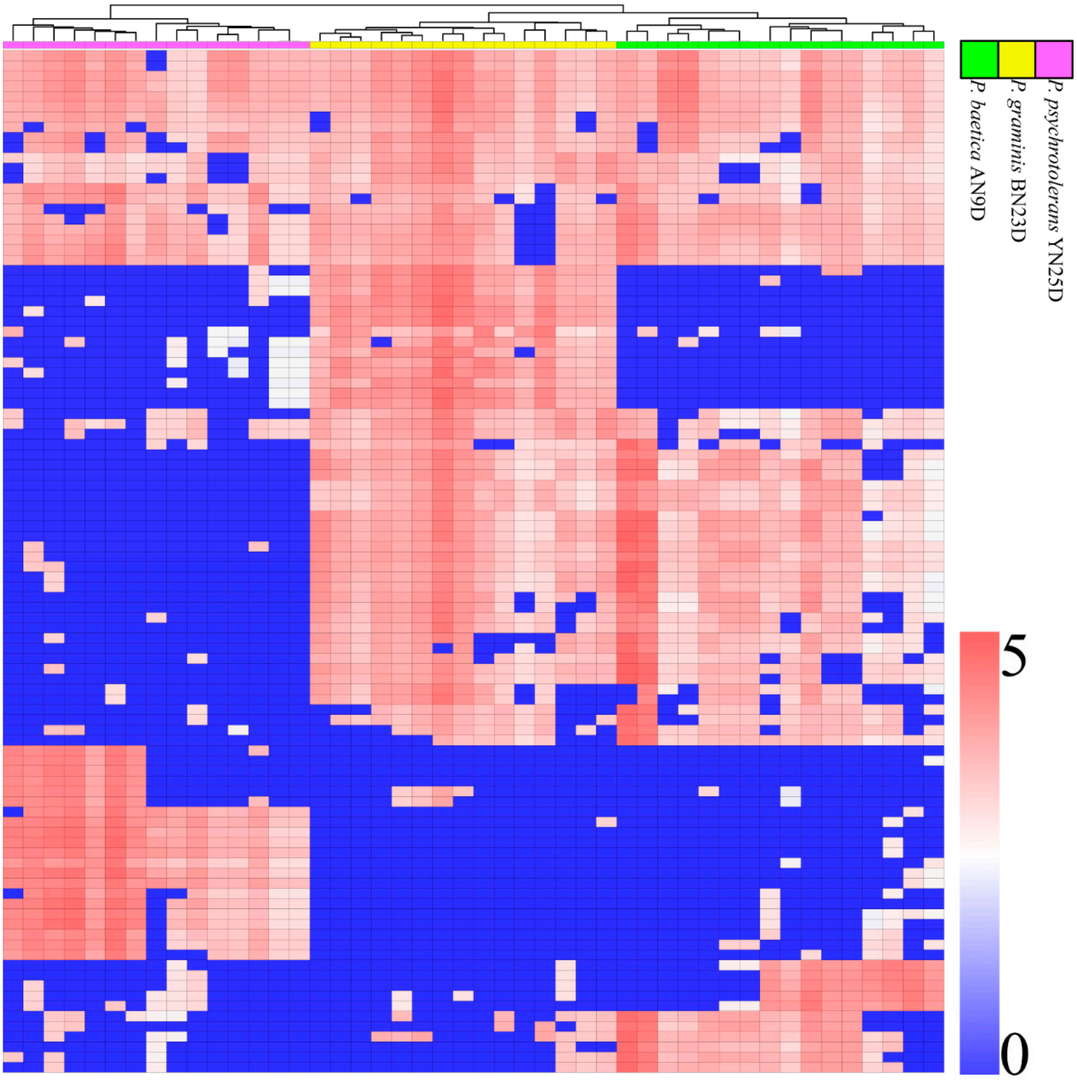

Figure S6. Clustering heatmap of the de-nosed and log10-convered data, the data including 15 biological repeat of *Pseudomonas baetica* AN9D, 15 biological repeat of *P. graminis* BN23D and 16 biological repeat of *P. psychrotolerans* YN25D.

Table S1. The strains and the isolation source

Strains	Source	Collection place
Microbacterium testaceum AR49	5-6 years old, the stem of *Populus tomentosa* Carrière	Shandong province, China
Pseudomonas koreensis AN12	5-6 years old, the stem of *Populus tomentosa* Carrière	Shandong province, China
Pseudomonas koreensis AN9	5-6 years old, the stem of *Populus tomentosa* Carrière	Shandong province, China
Pseudomonas koreensis AN11	5-6 years old, the stem of *Populus tomentosa* Carrière	Shandong province, China
Pseudomonas poae AN24	5-6 years old, the stem of *Populus tomentosa* Carrière	Shandong province, China
Pseudomonas poae AN4	5-6 years old, the stem of *Populus tomentosa* Carrière	Shandong province, China
Bacillus cereus BN77	1-2 years old, the stem of *Populus tomentosa* Carrière	Shandong province, China
Bacillus praedii BR25	1-2 years old, the stem of *Populus tomentosa* Carrière	Shandong province, China
Pseudomonas coleopterorum BN19	1-2 years old, the stem of *Populus tomentosa* Carrière	Shandong province, China
Pseudomonas coleopterorum BN21	1-2 years old, the stem of *Populus tomentosa* Carrière	Shandong province, China
Pseudomonas graminis BN23	1-2 years old, the stem of *Populus tomentosa* Carrière	Shandong province, China
Pseudomonas graminis BN24	1-2 years old, the stem of *Populus tomentosa* Carrière	Shandong province, China

Pseudomonas graminis BN57	1-2 years old, the stem of *Populus tomentosa* Carrière	Shandong province, China
Pseudomonas parafulva BN33	1-2 years old, the stem of *Populus tomentosa* Carrière	Shandong province, China
Pseudomonas parafulva BN52	1-2 years old, the stem of *Populus tomentosa* Carrière	Shandong province, China
Pseudomonas koreensis BN58	1-2 years old, the stem of *Populus tomentosa* Carrière	Shandong province, China
Pseudomonas koreensis BN60	1-2 years old, the stem of *Populus tomentosa* Carrière	Shandong province, China
Pseudomonas putida BN1	1-2 years old, the stem of *Populus tomentosa* Carrière	Shandong province, China
Pseudomonas putida BN11	1-2 years old, the stem of *Populus tomentosa* Carrière	Shandong province, China
Pseudomonas reidholzensis BN73	1-2 years old, the stem of *Populus tomentosa* Carrière	Shandong province, China
Bacillus cereus CN42	9-10 years old, the stem of *Populus tomentosa* Carrière	Shandong province, China
Bacillus cereus CN43	9-10 years old, the stem of *Populus tomentosa* Carrière	Shandong province, China
Microbacterium radiodurans CR16	9-10 years old, the stem of *Populus tomentosa* Carrière	Shandong province, China
Microbacterium testaceum CR19	9-10 years old, the stem of *Populus tomentosa* Carrière	Shandong province, China
Microbacterium testaceum CR36	9-10 years old, the stem of *Populus tomentosa* Carrière	Shandong province, China

Pseudomonas koreensis CR62	9-10 years old, the stem of *Populus tomentosa* Carrière	Shandong province, China
Pseudomonas koreensis CR63	9-10 years old, the stem of *Populus tomentosa* Carrière	Shandong province, China
Pseudomonas caspiana CN23	9-10 years old, the stem of *Populus tomentosa* Carrière	Shandong province, China
Pseudomonas caspiana CN39	9-10 years old, the stem of *Populus tomentosa* Carrière	Shandong province, China
Pseudomonas caspiana CR23	9-10 years old, the stem of *Populus tomentosa* Carrière	Shandong province, China
Pseudomonas caspiana CR51	9-10 years old, the stem of *Populus tomentosa* Carrière	Shandong province, China
Bacillus thuringiensis GN18	5-6 years old, the root of *Populus tomentosa* Carrière	Shandong province, China
Bacillus tropicus GN48-2	5-6 years old, the root of *Populus tomentosa* Carrière	Shandong province, China
Bacillus tropicus GN57	5-6 years old, the root of *Populus tomentosa* Carrière	Shandong province, China
Pseudomonas putida GN48	5-6 years old, the root of *Populus tomentosa* Carrière	Shandong province, China
Pseudomonas putida GN51	5-6 years old, the root of *Populus tomentosa* Carrière	Shandong province, China
Pseudomonas reidholzensis GN52	5-6 years old, the root of *Populus tomentosa* Carrière	Shandong province, China
Pseudomonas reidholzensis GN58	5-6 years old, the root of *Populus tomentosa* Carrière	Shandong province, China

Microbacterium oxydans HN76	5-6 years old, the stem of *Populus nigra* Linn. var. nigra	Shandong province, China
Pseudomonas coleopterorum HN11	5-6 years old, the stem of *Populus nigra* Linn. var. nigra	Shandong province, China
Pseudomonas coleopterorum HR4	5-6 years old, the stem of *Populus nigra* Linn. var. nigra	Shandong province, China
Pseudomonas koreensis HN21	5-6 years old, the stem of *Populus nigra* Linn. var. nigra	Shandong province, China
Pseudomonas putida HN40	5-6 years old, the stem of *Populus nigra* Linn. var. nigra	Shandong province, China
Pseudomonas canadensis HN10	5-6 years old, the stem of *Populus nigra* Linn. var. nigra	Shandong province, China
Pseudomonas canadensis HN57	5-6 years old, the stem of *Populus nigra* Linn. var. nigra	Shandong province, China
Rhizobium wenxiniae HN71	5-6 years old, the stem of *Populus nigra* Linn. var. nigra	Shandong province, China
Microbacterium oleivorans ON42	5-6 years old, the stem of *Populus canadensis* Moench	Shandong province, China
Microbacterium pumilum OR6	5-6 years old, the stem of *Populus canadensis* Moench	Shandong province, China
Pseudomonas koreensis OR28	5-6 years old, the stem of *Populus canadensis* Moench	Shandong province, China
Pseudomonas reidholzensis ON10	5-6 years old, the stem of *Populus canadensis* Moench	Shandong province, China
Pseudomonas reidholzensis ON46-4	5-6 years old, the stem of *Populus canadensis* Moench	Shandong province, China

Rhizobium cellulosilyticum ON47	5-6 years old, the stem of *Populus canadensis* Moench	Shandong province, China
Rhizobium wenxiniae ON46-1	5-6 years old, the stem of *Populus canadensis* Moench	Shandong province, China
Rhizobium zeae ON40	5-6 years old, the stem of *Populus canadensis* Moench	Shandong province, China
Rhizobium zeae ON51	5-6 years old, the stem of *Populus canadensis* Moench	Shandong province, China
Bacillus cereus YN8	5-6 years old, the leaf of *Populus tomentosa* Carrière	Shandong province, China
Pseudomonas putida YN18	5-6 years old, the leaf of *Populus tomentosa* Carrière	Shandong province, China
Pseudomonas putida YN9	5-6 years old, the leaf of *Populus tomentosa* Carrière	Shandong province, China
Pseudomonas reidholzensis YN16	5-6 years old, the leaf of *Populus tomentosa* Carrière	Shandong province, China
Pseudomonas reidholzensis YN19	5-6 years old, the leaf of *Populus tomentosa* Carrière	Shandong province, China
Bacillus infantis YN5	5-6 years old, the leaf of *Populus tomentosa* Carrière	Shandong province, China
